# Lateral center-edge angle in femoroacetabular impingement: from the sourcil or the rim of the acetabulum?

**DOI:** 10.1097/MD.0000000000040578

**Published:** 2024-11-22

**Authors:** Mustafa Çeltik, Onur Hapa, Selahaddin Aydemir, Eren Akin, Ahmet Kaan Arslan, Burak Duymaz, Onur Gürsan

**Affiliations:** a Department of Orthopedics and Traumatology, Ankara Oncology Research and Training Hospital, Ankara, Turkey; b Department of Orthopedics, Dokuz Eylül University Faculty of Medicine, Izmir, Turkey; c Department of Orthopedics, Kastamonu Research and Training Hospital, Kastamonu, Turkey.

**Keywords:** femoroacetabular impingement, LCEA, rim, sourcil

## Abstract

The correlation between clinical outcomes and preoperative/postoperative measures of the lateral center-edge angle (LCEA) will help establish the cutoff values for this measurement and determine whether to obtain it from the lateral acetabular rim (LCEA_R_) or the lateral end of the sourcil (LCEA_S_). The hypothesis was that the LCEA_S_ would be more sensitive than the LCEA_R_. An upper cutoff value of LCEA could predict better functional outcomes in FAI patients. 106 patients (103 unilateral, 3 bilateral) who underwent hip arthroscopy surgery due to FAI and had a minimum 2-year follow-up were included. Patient-reported outcomes included the mHHS and visual analogue scale for pain (Pain VAS). Radiological parameters (alpha angle, LCEA_S_, LCEA_R_) were evaluated at the pelvis, 45° Dunn X-rays. A receiver operating characteristic analysis was used to evaluate the correlation between significant variables and achievement of patient-acceptable symptomatic state (PASS) and calculate area under the curve (AUC) and critical values for LCEA. The mean age of the patients was 34 ±10 years with a mean follow-up of 5 years. There were 75 male and 31 female patients. The mHHS improved from 57 ± 11 before surgery to 93 ± 8 after surgery (*P* < .001). The Pain VAS decreased from 6 before surgery to 1 after surgery (*P* < .001). A higher frequency of reaching the PASS threshold for mHHS was associated with lower preoperative and postoperative _dunn_ LCEA_S_ and postoperative _dunn_ LCEA_R._ Preoperative _dunn_ LCEA_S_ ≤ 24.8° had an AUC of 0.67, predicting PASS (+) with 0.38 sensitivity and 0.93 specificity. Combining LCEA_S_ with other parameters further improved predictability. LCEA_S_ seems more predictive of clinical significance than LCEA_R_. However, predictivity exceeds the acceptable limit when they are measured together. The upper values for LCEA_S_ and LCEA_R_ seem to be 24° and 35°, respectively.

## 1. Introduction

The main morphology of femoroacetabular impingement (FAI) is cam type and pincer type. In pincer-type FAI, acetabular over-coverage predisposes impingement between the deep acetabular socket and the femoral neck, producing labral and articular cartilage damage.^[1]^ Hip arthroscopy provides successful treatment of both of these deformities.^[2]^ Lateral center-edge angle (LCEA) is a commonly used radiographic measure of pincer deformity and acetabular dysplasia. This angle helps to determine the degree of the acetabular deformity.^[3]^ It can be measured from the lateral edge of the acetabular rim^[4]^ or sourcil.^[5]^

Most studies consider 40° as the upper limit of normal for the LCEA and use the lateral edge of the *sourcil* as the reference point, following the technique described by Ogata et al.^[5]^ However, when measuring the LCEA angle, no significant differences were found between the over-coverage and normal coverage groups.^[3,6–8]^ Only the study by Chandrasekaran et al^[9]^ reported a lower magnitude of improvement in the over-coverage group (LCEA > 40°) compared to the control group (LCEA 25°–40°), which measured the LCEA using the technique described by Wiberg.^[4]^

Controversy also exists for borderline dysplasia. Studies describe borderline dysplasia as having LCEA lower than 25° and measuring it either from the lateral rim of the acetabulum^[10–12]^ or the lateral edge of the sourcil of the acetabulum.^[13]^ Meta-analyses and matched studies still did not find differences in functional results between borderline dysplasia and normal coverage (25°–40°) patient groups at short-term and long-term periods.^[14,15]^

The purpose of the present study was to clarify the effect of LCEA measured both from the lateral acetabular edge described by Wiberg^[4]^ and the lateral end of sourcil as described by Ogata et al^[5]^ preoperatively and postoperatively on clinically significant functional results of arthroscopically treated FAI patients after midterm follow-up.

The study hypothesizes that using the lateral edge of the sourcil as a reference point provides a better correlation with clinical outcomes than using the lateral edge of the rim. It also proposes an upper cutoff value for that measurement to predict clinically significant and/or better functional results for FAI patients.

## 2. Materials and methods

The present study included 106 patients who underwent hip arthroscopy due to FAI and had a minimum of 2 years of follow-up. Local Institutional Review Board approval was obtained from the Dokuz Eylül University Non-Interventional Research Ethics Committee. FAI was diagnosed based on clinical symptoms and radiographic findings (alpha angle > 50° for cam deformity using the 45° Dunn view,^[16,17]^ LCEA > 40° for pincer deformity^[4,9,18]^). Patients were excluded if they had avascular necrosis, advanced-level hip osteoarthritis (Tönnis ¨Grade > 1),^[19]^ any previous ipsilateral hip surgery, revision hip arthroscopy or incomplete radiographs or hip dysplasia (LCEA < 20°).^[4,5]^

### 2.1. Surgical procedure

The patient is placed supine on a hip arthroscopy–specific traction table to obtain appropriate hip distraction against a well-padded perineal post. A horizontal interportal capsulotomy improves the visualization and access to the central compartment. A 4.5-mm arthroscopic burr is used to perform acetabuloplasty. Degenerative labral tears or those with multiple cleavage planes were considered irreparable, and unstable flaps were selectively debrided. Tears that involved the base of the labrum with chondrolabral disruption were repaired using 1 to 3 suture anchors. Traction is then released, the peripheral compartment is entered, and decompression of the cam deformity is performed and confirmed by intraoperative fluoroscopy and arthroscopic dynamic examination. The capsule was routinely left open at the end of the procedure.

### 2.2. Rehabilitation

All patients were instructed to use crutches to limit weight for 2 weeks. Daily passive range of motion exercises were begun on the first postoperative day. At 3 weeks, active range of motion and full weight-bearing were commenced. After 6 weeks, strengthening and light treadmill walking were begun. For the first 4 weeks, daily oral anti-inflammatory medication was prescribed.

We collected data on continuous and categorical demographic and clinical variables, including age, sex, body mass index, and follow-up time.

**Figure 1.**
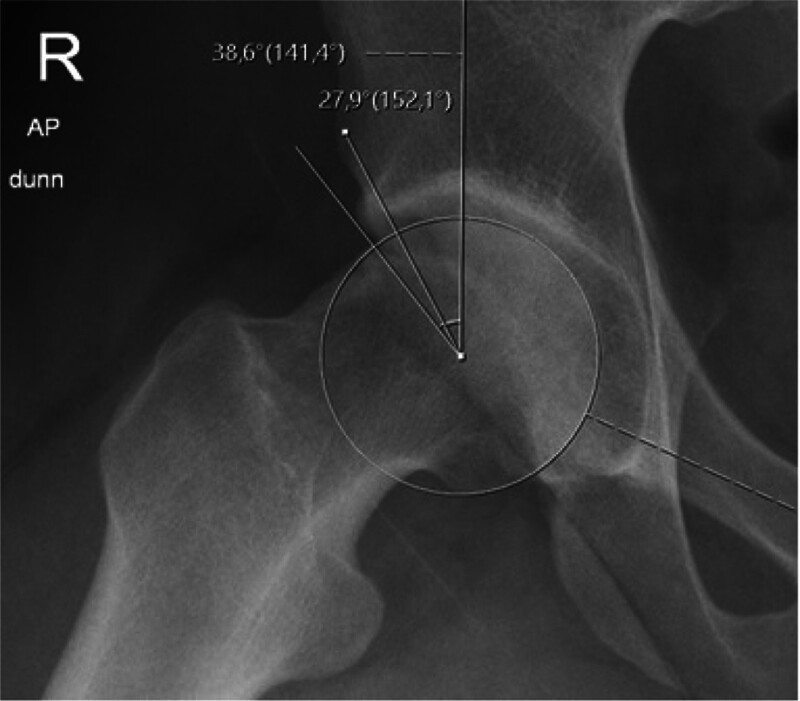
LCEA_S_ “sourcil” and LCEA_R_ “rim” measurement techniques.

Radiographs were obtained and evaluated by consensus with senior surgeons for all patients using the anteroposterior supine pelvis, Dunn 45° X-rays. Osteoarthritis was graded using the Tönnis ¨classification at preoperativë and last follow-up pelvis X-rays.^[19]^ LCEA was measured using the methods described by Wiberg^[4]^ “LCEA_R_” and Ogata^[5]^ “LCEA_S_” on pelvis X-rays and Dunn 45° X-rays (Fig. 1). The LCEA was measured on the Dunn 45° X-rays, explicitly focusing on the anteroposterior (AP) view of the hip with the femur in flexion and abduction, which may result in pelvic tilt and rotation. However, the literature indicates that the LCEA remains accurate and unaffected by pelvic tilt and rotation, ensuring the evaluation is reliable and feasible.^[20,21]^ The alpha angle was measured preoperative and early postoperative Dunn 45° views.^[16,17]^

The patient-reported outcomes, including the modified Harris Hip Score (mHHS)^[22]^ and Visual Analog Scale for Pain (Pain VAS), were collected by contacting the patients directly. Pain VAS and the mHHS were recorded the day before the surgery and at the last follow-up assessment. Scores beyond 83 points for mHHS indicate a patient-acceptable symptomatic state (PASS) threshold or improved function within 5 years postoperatively.^[23,24]^ The minimal clinically important difference was not used because patients had been assessed at different time intervals.

Statistical analysis Data analysis was performed using SPSS version 24 for Windows (IBM, SPSS statistics). Values of *P* < .05 were considered statistically significant. The effect of dichotomous or Categorical variables, including gender, labrum treatment (debridement or repair), body mass index, and follow-up time, on whether or not reaching the PASS were analyzed using the Fisher exact or chi-square test. Continuous variables like (age and alpha angles) were analyzed using the Mann–Whitney U test. Preop to postop changes for VAS and mHHS were assessed using the Wilcoxon test.

The receiver operating characteristic curve (ROC) and area under the curve (AUC) were analyzed to further determine discriminatory threshold values for LCEA_S_ and LCEA_R_ and the frequency of reaching PASS values for mHHS. An AUC between 0.70 and 0.80 was reported to be acceptable discrimination.

## 3. Results

The mean age of the patients was 34±10 years (24–54). The mean follow-up was 5±2 years (3–7). There were 75 male and 31 female patients. Latest mHHS were higher (93±8) and VAS score lower (1±1) compared to preoperative values (57±11, 6±1, *P* < .001)

Patient demographics and comparisons, including LCEA measurements between PASS(+) and PASS (−), were made in Tables 1 and 2. PASS (+) had lower preoperative and postoperative_dunn_ LCEA_S_ and postoperative_dunn_ LCEA_R_ compared to PASS (−) patients (*P* < .05). Dunn LCEA measurements were not different than pelvis X-ray LCEA measurements (*P* > .05). ROC analysis showed that preoperative_dunn_ and postoperative _dunn_ LCEA_S_ and postoperative _dunn_ LCEA_R_ exhibited statistical significance in identifying PASS (+) patients. Pre-operative_pelvis_ LCEA_S_ approached statistical significance (*P* = .06).

**Table 1:** Patient demographics and comparisons between PASS (+) and PASS (−) patients. *P* < .05 statistically significant.

Patients (n: 106) Mean (95% CI)	PASS (+) N: 94	PASS (−) N: 15	*P* value
Age	33 (25–42)	36 (32–51)	.068
Follow-up (yr)	4 (3–6)	5 (3–6)	.8
Symptoms duration (months)	12 (9–20)	12 (6–19).	.3
Sex	69M, 25F	9M, 6F	.3
Tönnis grade	63 (0), 31 (I)	9 (0), 6 (I)	.5
Active smokers	44	7	.9
BMI	25 (23–27)	27 (24–29)	.2
Labrum debride or repair	17/77	5/10	.1
Preoperative α°	80 (77–84)	79 (76–81)	.2
Postoperative α°	48.7 (45.8, 52.2)	48.1 (45.1, 49.7)	.2

**Table 2:** LCEA_S_ and LCEA_R_ comparisons between PASS (+) and PASS (−) patients.

LCEA_S_, LCEA_R_	PASS (+)	PASS (−)	*P* value
LCEA_S_			
Preop			
Pelvis	25 (23–30)	28 (25–33)	.1
Dunn	26 (23–30)	29 (26–32)	.03*
Postop			
Pelvis	25 (22–29)	28 (24–31)	.1
Dunn	25 (22–29)	29 (25–31)	.03*
LCEA_R_			
Preop			
Pelvis	38 (34–44)	39 (36–43)	.7
Dunn	39 (34–44)	42 (37–45)	.1
Postop			
Pelvis	37 (32–43)	39 (35–42)	.2
Dunn	37 (33–42)	40 (37–44)	.04*

* Values are statistically significant.

**Figure 2.**
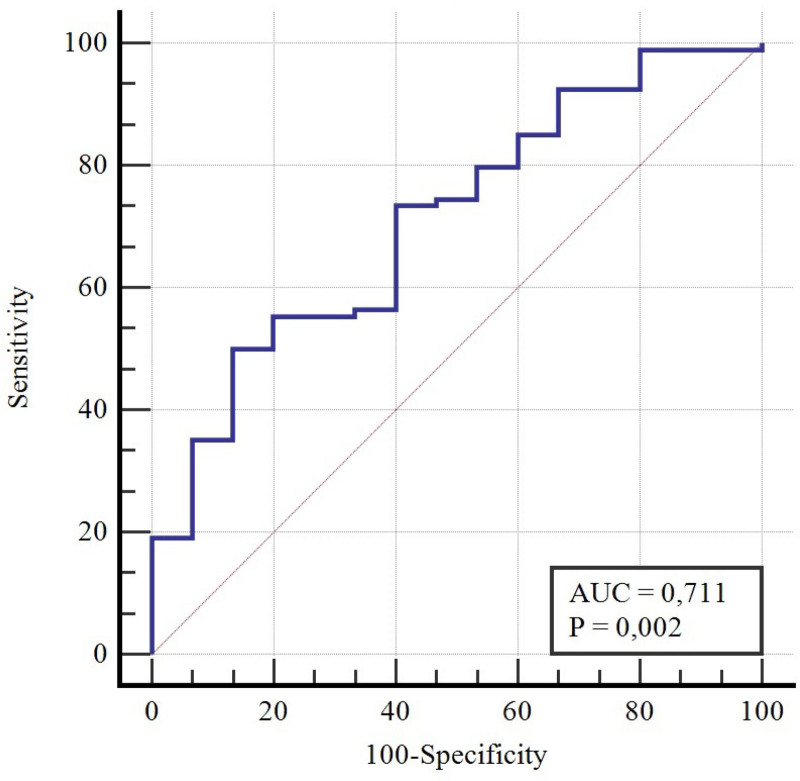
ROC analysis of Preoperative_dunn_ LCEA_S_ combined with Preop_Pelvis_ LCEA_S_, Preop_dunn_ LCEA_R,_ Preop_pelvis_ LCEA_R_.

However, all had AUC lower than 0.7. AUC exceeded the threshold value of 0.7 when Preoperative _dunn_ LCEA_S_ was evaluated with 3 other preoperative measurements (Table 3, *P* < .05, Fig. 2).

**Table 3:** ROC analysis of LCEA_S_ and LCEA_R_.

	Cutoff value	Sensitivity	Specificity	AUC	*P* value
Preop_dunn_ LCEA_S_ (1)	≤24.8	0.38	0.93	0.67	.01*
Preop_Pelvis_ LCEA_S_ (2)	≤25.8	0.53	0.73	0.63	.06
Preop_dunn_ LCEA_R_ (3)	≤36	0.36	0.93	0.61	.09
Preop_pelvis_ LCEA_R_ (4)	≤35.1	0.34	0.93	0.52	.6
1 + 2 + 3 + 4	≤25.7	0.50	0.86	0.71	.002*
Postop_dunn_ LCEA_S_	≤23.8	0.40	0.93	0.67	.01*
Postop_dunn_ LCEA_R_	≤35.5	0.44	0.93	0.66	.02*

* Statistically significant *P* < 0.05.

## 4. Discussion

In summary, the study found that measuring the LCEA from the sourcil (as described by Ogata et al^[5]^) is more predictive than using the measurement from the lateral acetabular rim (as described by Wiberg^[4]^) to detect clinical significance in functional scores, yet still lower than the accepted threshold for ROC analysis. However, when combined, the predictability exceeds the accepted threshold for ROC analysis (AUC > 0.7). The upper values of the LCEA sourcil and the lateral rim were lower than 25° and 36°, respectively.

Among 3 studies measuring LCEA from the sourcil, 2 reported over-coverage as >40°, the third as ≥39°, and none reported any difference between the normal coverage group (>25°) and over-coverage groups.^[6–8]^ One study measuring LCEA_S_ at postoperative X-rays instead of preoperative cited 25° to 35° as normal coverage and >35° as borderline over-coverage and reported no difference between functional scores post arthroscopy or rates of conversion to total hip arthroplasty while there was more revision at 25° to 35° group compared to over-coverage and borderline dysplasia (20°–24.9°) groups.^[3]^

Two studies reported the outcomes using the LCEA_R_, the first study by Chandrasekaran et al^[9]^ reported a lower magnitude of improvement in the over-coverage group (LCEA > 40° + profunda acetabulae) compared to the normal coverage group (25°–40°) in the short term, while the other study by Kingery et al^[18]^ reported no difference in functional outcome or rate of revision at midterm between global over-coverage (LCEA_R_ ≥ 40°, with coxa profunda), lateral over-coverage (LCEA_R_ ≥ 40°, without coxa profunda), and no over-coverage (LCEA < 40°).^[18]^

For borderline dysplasia, studies cite LCEA as lower than 25° and measure it as described by Wiberg et al^[4,10–12]^or Ogata et al.^[5,13]^ They report no differences for all outcomes compared to normal coverage in both the short and long term.^[14,15]^

The present study found stronger LCEA_S_ predictability than LCEA_R_ at comparison and ROC analyses, although AUC approached the acceptable threshold while passed at combined measurement. This could be due to the fact that the acetabular coverages differ between the landmarks from which the LCEA measurements are obtained. LCEA_S_ represents the antero-superior acetabular coverage rather than the supero-lateral coverage presented at LCEA_R_.^[25]^

There are some limitations. First, only the mHHS and Pain VAS were used as patient-reported outcomes, and other outcome scores, like hip outcome scores or the International Hip Outcome Tool-12, were not used. Second, the severity of chondrolabral lesions was not graded, though we considered the type of labrum treatment and measured indirect signs of cartilage damage, such as Tönnis grading, sex, and preoperative alpha angle.^[26–29]^

Third, the present study is a single-surgeon case series study without a control group and adjustment of confounding factors and with retrospective analysis of prospectively gathered data. Fourth, although measurements were done by consensus with senior surgeons, interobserver or intraobserver reliability was not investigated. Lastly, although preop LCEA_S_ pelvis X-ray measurements approached statistical significance, and the literature suggests that the LCEA measurement did not differ between hip or pelvis X-rays, the present study found the main differences at Dunn X-ray, possibly due to minor measurement differences.

In conclusion, LCEA measured from the sourcil seems more predictive for clinically significant improvement; however, it is still under the accepted threshold, which is overcome by co-measurement with LCEA_R_. LCEA_S_ and LCEA_R_ should be below 25° and 36°, respectively.

## Author contributions

**Conceptualization:** Onur Hapa, Burak Duymaz, Onur Gürsan.

**Data curation:** Onur Hapa.

**Formal analysis:** Mustafa Çeltik, Ahmet Kaan Arslan.

**Funding acquisition:** Mustafa Çeltik.

**Investigation:** Selahaddin Aydemir.

**Methodology:** Mustafa Çeltik, Onur Hapa, Selahaddin Aydemir, Burak Duymaz.

**Project administration:** Mustafa Çeltik, Selahaddin Aydemir, Burak Duymaz.

**Resources:** Selahaddin Aydemir, Ahmet Kaan Arslan, Onur Gürsan.

**Supervision:** Onur Hapa, Selahaddin Aydemir, Onur Gürsan.

**Validation:** Eren Akin, Burak Duymaz.

**Visualization:** Eren Akin.

**Writing—original draft:** Mustafa Çeltik, Onur Hapa, Eren Akin, Onur Gürsan.

**Writing—review & editing:** Mustafa Çeltik, Onur Hapa, Ahmet Kaan Arslan.
